# Effects of curcumin supplementation on abdominal surgical wound healing

**DOI:** 10.1590/acb392124

**Published:** 2024-04-15

**Authors:** Melquesedeque dos Santos, Eurico Cleto Ribeiro de Campos, Rivair Gonçalves, Adriana Yuriko Koga, Pedro Afonso Kono, Matheus Von Jelita Salina, Elder Dalazoana, Alceu de Oliveira Toledo, Leandro Cavalcante Lipinski

**Affiliations:** 1 Universidade Estadual de Ponta Grossa – Departamento de Medicina – Ponta Grossa (PR), Brazil.; 2 Faculdade Evangélica Mackenzie – Departamento de Oncologia – Curitiba (PR), Brazil.; 3 Universidade Estadual de Ponta Grossa – Departamento de Farmácia – Ponta Grossa (PR), Brazil.

**Keywords:** Curcumin, Surgical Wound, Wound Healing, Rats, Wistar

## Abstract

**Purpose:**

To evaluate the effects of curcumin supplementation on abdominal surgical wound healing in rats using clinical, histological, and hematological parameters.

**Methods:**

Forty Wistar rats were randomly divided into two groups: the curcumin group, and the control group. The curcumin group received, in addition to water and standard feed, curcumin via gavage at the dose of 200 mg/kg for seven days preceding and seven days following surgery. The control group received only water and standard feed. Both groups underwent median laparotomy and left colotomy. On the eighth postoperative day, the groups were euthanized, and the left colon was resected for histological analysis.

**Results:**

In the preoperative evaluation, there was a significant decrease in the mean C-reactive protein levels in the curcumin group (0.06) compared to the control group (0.112) (p = 0.0001). In the postoperative wound healing assessment, a significant decrease was observed in inflammatory infiltrate (p = 0.0006) and blood vessel count (p = 0.0002) in the curcumin group compared to the control group.

**Conclusions:**

Curcumin supplementation was able to significantly reduce inflammatory parameters in both pre-and post-operative phases of abdominal surgical wounds in rats.

## Introduction

Operative wounds are classified as acute and intentionally created to prevent potential complications. However, they can also evolve in a complex manner and be considered chronic when they require a prolonged healing time. Additionally, there are different types of wound healing, categorized as primary or secondary intention. Primary intention occurs when there is no approximation of the wound edges, leading to a slower healing process. Various factors can alter the type of wound healing and pose risk factors, ranging from the patient’s own conditions to the type of surgical technique adopted. Notably, complications such as infection and dehiscence are more frequently observed^1^.

Wound healing is the natural process of tissue recovery following surgical trauma. It comprises three main phases: the inflammatory phase, fibroplasia, and maturation^2^.

The initial inflammatory phase is characterized by vasoconstriction, resulting from the release of inflammatory mediators, such as pro-inflammatory cytokines and thromboxane A2. These mediators are released by platelets adhering to the damaged and exposed endothelium. After local and natural hemostasis control, prostaglandins are released, promoting vasodilation and the migration of chemotactic molecules, attracting neutrophils and macrophages to the site of surgical trauma. Through the action of macrophages, devitalized tissues are removed, initiating the proliferation phase^2^^,^ 3.

The cellular proliferation phase is a continuation of the inflammatory phase, lasting approximately 48 h to one week. Angiogenesis, fibroblast migration, stimulated by chemotactic factors released by macrophages, and epithelialization predominate in this phase. With the formation of blood vessels, originating from the migration of endothelial cells from vascular sprouts, the fibroplasia phase begins, with fibroblasts and extracellular matrix production, primarily collagen, predominating. This phase can extend from the seventh day up to three weeks. It is one of the most crucial phases, as the quantity and quality of deposited matrix will influence the scar, as well as the extension or cessation of the inflammatory phase^2^^,^ 3.

The final phase is maturation, in which the quantity of fibroblasts is reduced, and myofibroblasts predominate, leading to matrix contraction, resulting from bridges between collagen fibers and the formation of a mature scar^4^.

Regarding the healing process of intestinal anastomoses, fibroblasts begin to emerge during the advanced inflammatory phase and become the predominant cellular type in the wound area by the fourth day, with the proliferation phase usually extending for approximately 14 days. It is also important to differentiate that the gastrointestinal tract has differences compared to the skin, such as a broader and more diverse microbiota, which can alter the healing period^5^.

Given this entire process, the use of curcumin is a potential aid in wound contraction, as it accelerates the phases of the healing process6. Its anti-inflammatory action is attributed to its ability to reduce inflammatory pathways, such as interleukin (IL)-1, IL-6, and transcription factors of protein I and nuclear factor kappa B (NfkB). During the proliferative phase, it is capable of increasing reepithelialization speed, as well as granulation tissue and the amount of type III collagen. In the remodeling process, there is an increase in the production of extracellular matrix components and the concentration of transforming growth factor-β (TGF-β) in the wound area, which, in turn, contributes to wound contraction^7^.

Corroborating the potential effects on abdominal surgical wound healing, through the regulation of inflammatory signaling pathways and inhibition of inflammatory mediator synthesis, benefits in different tissues of the human body have already been demonstrated^8^. Research has shown that curcumin, due to its intrinsic properties, confers benefits in managing different inflammatory conditions, such as arthritis, psoriasis, atherosclerosis, and the systemic impacts of COVID-19^9^^–^ 11.

Based on this, this study aimed to analyze the supplementation of curcumin and, particularly, its effect on the wound healing process, evaluating hematological and clinical parameters post-left colectomy in Wistar rats.

## Methods

This was an experimental study conducted at the Laboratory of Operative Technique, Prof. Gilberto Luis Ortolan, at the Universidade Estadual de Ponta Grossa, between the years 2022 and 2023. The research was conducted with approval from the Ethics Committee for Animal Use and the Brazilian Directive for Care and Use of Animals for Scientific and Educational Purposes, established by the National Council for Animal Experimentation, and followed international standards for animal experimentation (Protocol no. 23.000011573-2).

Forty male Wistar rats (*Rattus norvegicus albinus*, Rodentia, Mammalia) of the same lineage, weighing between 230 and 338 g, were used. These animals were obtained from the Animal Facility of the Universidade Estadual de Ponta Grossa and were housed in groups of four per cage in a climate-controlled environment (22 ± 1ºC) with controlled lighting. Throughout the experiment, they had *ad libitum* access to water and food.

Curcumin, the nutrient of interest, was supplemented via gavage in the pre- and post-operative period to the experimental group, seven days prior and seven days after the surgical procedure, in addition to their regular water and food. The concentration of curcumin was 200 mg/mL, administered through a size 8F gastric gavage tube, with the length of 10 cm and internal diameter of 2 mm. To formulate the curcumin solution administered via gavage, we used 1,560 mg of turmeric, with the concentration of 96% curcumin, diluted in 5 mL of glycerin and 10 mL of 0.9% saline solution. This resulted in a solution of 100 mg/mL.

All rats underwent a 12-h fasting period before the surgical procedure. This was done to minimize the presence of fecal content in the colon, making the surgical procedure easier and reducing the risks of contamination and adhesion formation. For anesthesia, intraperitoneal administration of ketamine at the dose of 40 mg/kg and xylazine at the dose of 10 mg/kg was used.

**Figure 1:**
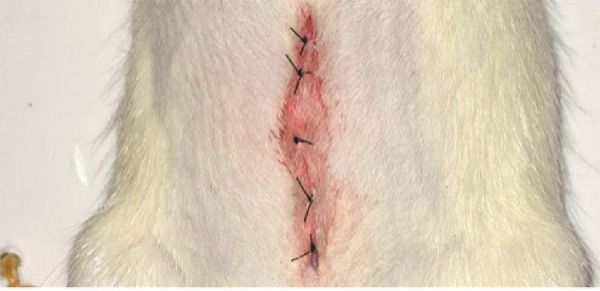
Closure of the abdominal wall with simple suture.

Source: Elaborated by the authors.

After anesthesia, the animals were weighed. Subsequently, trichotomy of the middle abdominal region was performed, and the animals were placed on the surgical table with their limbs extended. An approximately 3-cm long midline abdominal incision provided access to the abdominal cavity for exposure of the colon. The colon was sectioned 1.5 cm, at the level of the transverse and descending colon, with preservation of the colic vessels. Reconstruction was achieved by termino-terminal anastomosis in a single layer with two separate points of monofilament nylon thread, 5 cm apart. The abdominal wall was closed with anchored continuous suture of monofilament nylon 4.0 (Fig. 1) in two planes: muscle-aponeurotic, and intradermal.

After anesthetic recovery, the animals were isolated in cages and resumed *ad libitum* access to water and food 1 h after the procedure.

On the first postoperative day, curcumin administration was resumed. The rats were euthanized on the eighth postoperative day using an overdose of anesthetic with ketamine (160 mg/kg) and xylazine (40 mg/kg). For animal weighing, the Marte model AM 5500 scale, calibrated according to the National Institute of Metrology, Quality and Technology (INMETRO) standards, was used. Subsequently, 5 mL of blood was obtained from each specimen for biochemical analysis through a median thoracotomy and cardiac puncture in the right ventricle. After this step, the segment of the colon that underwent surgery was immersed in a 10% formalin solution for fixation, to be further used for histological study.

**Figure 2:**
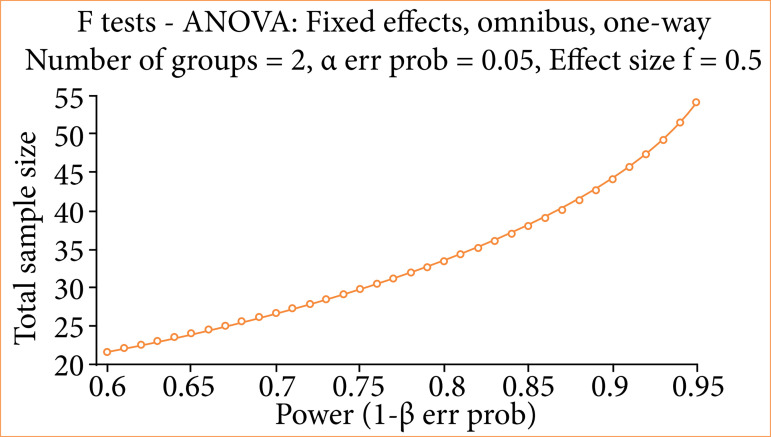
Sample size calculation in GPower 3.1.9.2.

Source: Elaborated the authors.

Statistical analysis was performed using GraphPad Prism 6, employing one-way analysis of variance (ANOVA) for multiple comparisons, followed by Tukey’s test, with the confidence interval of 95% (p ≤ 0.05). Sample size was calculated adopting a significance level of α = 0.05, for two groups of 20 animals each. The calculations were based on mean and standard deviation, establishing a test power of 90%, using the GPower 3.1.9.2 program (Fig. 2).

Regarding postoperative evolution, the following aspects were evaluated: weight gain or loss, occurrence of postoperative mortality, and wound analysis. Hematocrit and C-reactive protein (CRP) levels were assessed. For this purpose, blood was collected by puncturing the vena cava during surgery and by cardiac puncture after euthanasia. Hematocrit measurement was performed by filling a capillary tube with blood up to 3/4 of its height, with one end occluded with appropriate clay. The tube was then placed in a centrifuge adjusted for 5 min at 10,000 rpm. The result was interpreted on a reading scale by observing the separation limit between erythrocyte mass and plasma.

Finally, serum was obtained by centrifuging 2 mL of blood in a refrigerated centrifuge (model NT 816 Novatechnica) for 5 min at 4,000 rpm and kept refrigerated at -20ºC until the time of analysis. Quantitative determination of CRP was performed by automated turbidimetry (selectra XL). The analytical principle was the turbidimetric method with enhanced latex. The presence of ≥ 5 mg of CRP in the sample causes agglutination of latex particles coated with anti-CRP antibodies. The degree of agglutination is proportional to the concentration of CRP in the sample. This agglutination process is based on the optical detection of very small particles suspended in liquid, forming immune complexes. The dilution becomes turbid, which is proportional to the number of antigens present in the sample.

The quantification of inflammatory cells and vessels was performed using ImageJ software–cell counter with the counting of three standardized quadrants per image. The images were obtained with an Olympus AX70 microscope, with a polarizer. To avoid variations related to the capture process, the camera settings and light intensity were identical for all samples. The images were analyzed using the ImageJ program, and the results were expressed in pixels.

**Figure 3:**
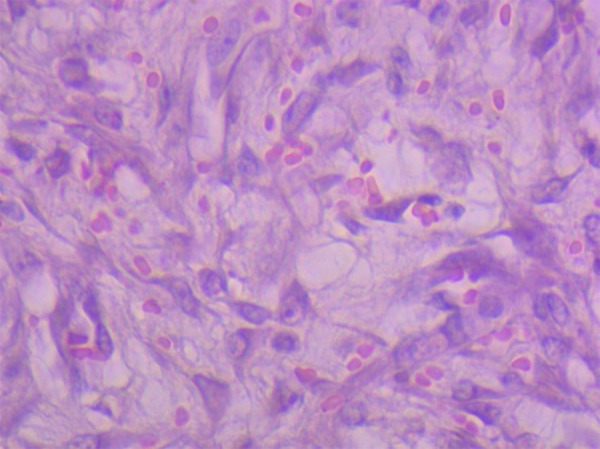
Standardized quadrant counting model image.

Source: Elaborated by the authors.

For the histological study (Fig. 3), the segment of the colon that underwent surgery was used. The samples were immersed in a 10% formalin solution for fixation. After this step, dehydration, diaphanization, and tissue embedding were performed. The blocks were subjected to the microtomy process, resulting in cuts with thickness of 5 μm. These sections were stained with hematoxylin and eosin. Images of the histological slides were acquired blindly to avoid bias.

## Results

Out of 40 operated rats, three deaths occurred in the control group and five in the curcumin group. Among four rats, two from the control group and two from the curcumin group, it was not possible to analyze the results due to intraoperative death. Therefore, the control group consisted of 17 rats (53.1%), and the curcumin group consisted of 15 rats (46.8%).

The average weight of the rats was 266 g with median of 262 g, ranging from a minimum of 210 g to a maximum of 338 g (Table 1). As for hematocrit, the mean value was 48.35 preoperatively and 43.5 postoperatively. In the curcumin group, the mean was 47.26 preoperatively and 42.53 postoperatively. In the control group, the mean was 49.41 preoperatively and 44.47 postoperatively, without achieving statistical significance.

**Table 1:** Describing weight and mortality variables, showing group homogeneity.

Variable	Total n (%)	Control n (%)	Curcumin n (%)	P-value
Death	8 (17.39)	3 (37.5)	5 (21.74)	0.3497*
**Variable**	**Total n (%)**	**Control n (%)**	**Curcumin n (%)**	**P-value**
	**Mean/median (SD); range**	**Mean/median (SD); range**	**Mean/median (SD); range**	
Preoperative weight	262.4 (26.6); 216–326	261.1 (29.4); 216–326	263.8 (24.3); 232–314	0.753**
Postoperative weight	270.3 (32.3); 210–338	267.7 (31.1); 226–328	273.2 (34.6); 210–228	0.636**
Preoperative CRP	0.094 (0.027); 0.06–0.17	0.112 (0.025); 0.09–0.17	0.076 (0.014); 0.09–0.1	0.0001***
Postoperative CRP	0.06 (0.01); 0.05–0.1	0.06 (0.01); 0.05–0.09	0.06 (0.011); 0.05–0.1	0.7861***

CRP: C-reactive protein; SD: standard deviation;

* Fisher’s exact test;

** Student’s t-test;

*** Mann-Whitney test.

Source: Elaborated by the authors.

For CRP, the average value preoperatively was 0.094 and postoperatively it was 0.06. Comparing the two groups preoperatively, the average CRP of the control group was 0.112 and in the curcumin group it was 0.06, obtaining statistical significance (p = 0.0001). When comparing the postoperative CRP results, no statistical significance was obtained (p = 0.7861) (Table 1).

**Figure 4:**
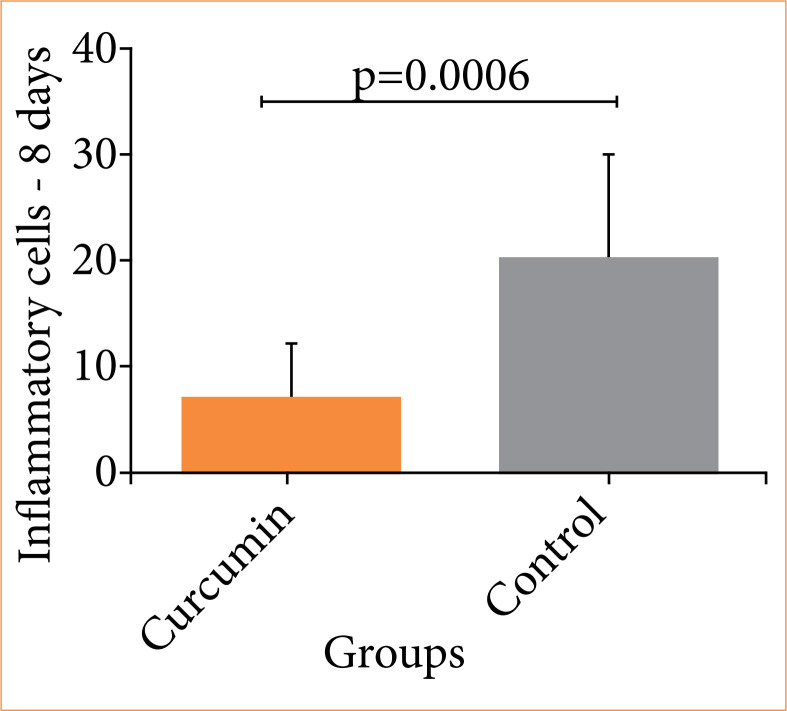
Comparison of the quantity of inflammatory cells in the colon between the curcumin and control groups (Student’s t-test).

Source: Elaborated by the authors.

**Figure 5:**
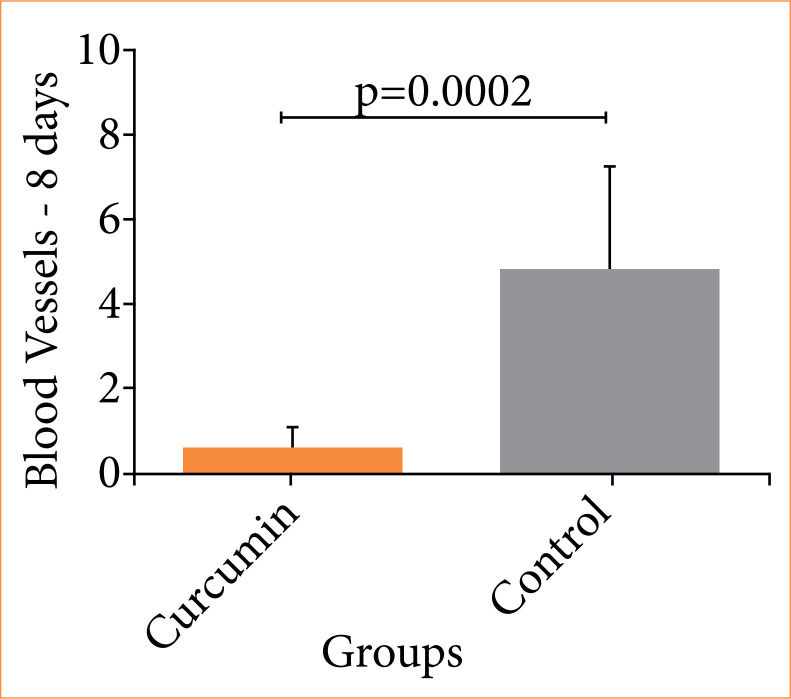
Comparison of the quantity of blood vessels in the colon between the curcumin and control groups (Student’s t-test).

Source: Elaborated by the authors.

**Figure 6:**
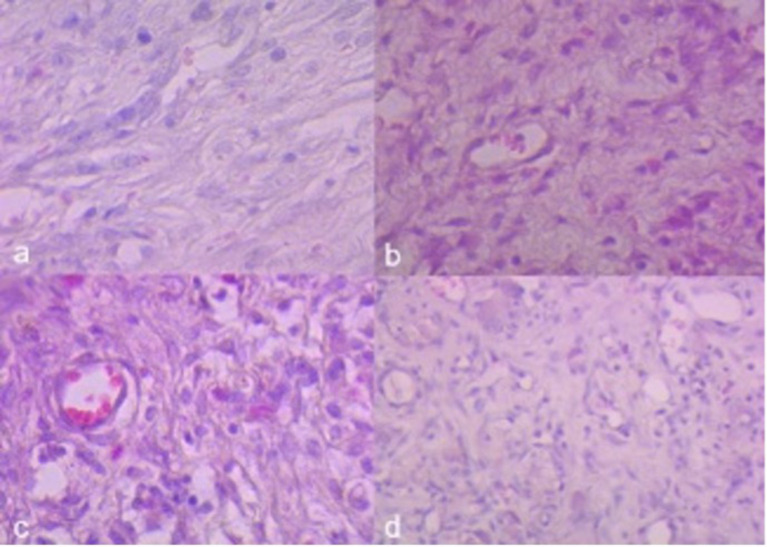
Histological examination of colon segments from both the curcumin and control groups utilizing hematoxylin-eosin staining. (a and b) Curcumin group presented lower inflammatory infiltrate, number of inflammatory cells, and less blood vessels compared to (c and d) the control group.

Source: Elaborated by the authors.

In the evaluation of postoperative wound healing, a significant decrease in inflammatory infiltrate (p = 0.0006) and blood vessel count (p = 0.0002) was found in the curcumin group compared to the control group (Figs. 4, 5, and 6).

## Discussion

Surgical procedures involving the left colon have been shown to have more complications compared to those on the right side. This is attributed to anatomical differences, bacterial proliferation, the surgery itself, or other factors. Additionally, the arterial vascularization of both colons differs, resulting in different blood flow, which is greater in the right colon^12^^,^ 13. Thus, the healing process may occur differently due to the specific characteristics of each colon.

Postoperative wound complications are a significant cause of surgical complications. Marques et al.^14^ sought to assess the risk of wound dehiscence following surgery, concluding that impaired wound healing led to longer intensive care unit and hospital stays, as well as difficulties in mobility and bedside rest.

Numerous scientific studies have demonstrated the anti-inflammatory and antioxidant properties of curcumin, resulting in the reduction of free radicals and optimization of wound healing^15^^,^ 16. In the present study, oral administration of curcumin via gavage demonstrated effectiveness in reducing blood vessels compared to the control group. However, it is worth noticing that the bioavailability of curcumin and its effects is lower when administered orally compared to topically^17^^,^ 18.

The use of proteins as biomarkers for systemic inflammatory response is well established, with CRP being prominent due to its ease of acquisition, cost-effectiveness, and extensive literature support^19^^,^ 20. CRP levels can rise rapidly in response to acute inflammatory conditions^21^.

In this study, a statistically significant difference was observed in CRP levels between the groups preoperatively (p < 0.05). However, in the postoperative period, no statistically significant differences were identified (p > 0.05). The nonsignificant difference in postoperative CRP may be attributed to factors such as timing, dosage, and even the surgical procedure itself. In the preoperative period, however, CRP has proven to be a response to any tissue alteration, involving complex changes spanning from the endocrine, hematologic, immune, to the neurological systems. Thus, even emotional factors may be involved in the statistically significant preoperative value obtained^22^.

Regarding clinical parameters, some studies suggest that curcumin may reduce weight gain in response to increased peripheral insulin sensitivity^23^^–^ 25. However, in this study, no significant variation in weights was observed. Further comprehensive and definitive studies are warranted to explore the potential of curcumin, a promising natural bioactive, as an adjunct in combating obesity.

In this study, it was observed that Wistar rats supplemented with curcumin exhibited a significant reduction in inflammatory infiltrates and blood vessels. Additionally, there was a significant reduction in preoperative CRP levels, indicating a decrease in acute response.

For future studies, we suggest increasing the number of animals, as well as exploring variations in dosage and different methodologies, considering that curcumin has various forms of bioavailability, and topical application could be highly beneficial in the healing process. Nevertheless, further research is needed to address any remaining uncertainties.

## Conclusion

Oral curcumin supplementation was able to significantly reduce inflammatory parameters in both preoperative phase (CRP) and postoperative phase (inflammatory infiltrate and blood vessel count) in abdominal surgical wounds of Wistar rats.

## Data Availability

All data sets were generated or analyzed in the current study.
